# Phenolic Profile and Antioxidant Activity of Fractions of Procyanidin-Rich Hawthorn (*Crataegus monogyna* Jacq.) Bark Extract Separated by Low-Pressure Liquid Chromatography

**DOI:** 10.3390/molecules30224375

**Published:** 2025-11-12

**Authors:** Magdalena Karamać, Michał A. Janiak, Katarzyna Sulewska, Ryszard Amarowicz

**Affiliations:** Team of Chemical and Physical Properties of Food, Institute of Animal Reproduction and Food Research, Polish Academy of Sciences, Trylińskiego 18 Str., 10-683 Olsztyn, Poland; m.janiak@pan.olsztyn.pl (M.A.J.); k.sulewska@pan.olsztyn.pl (K.S.); r.amarowicz@pan.olsztyn.pl (R.A.)

**Keywords:** single-seeded hawthorn, condensed tannins, column chromatography, phenolic profile, antiradical activity, reducing power, emulsion oxidation

## Abstract

Plant materials rich in proanthocyanidins are fractionated to determine the structure of these compounds and relate it to bioactivity. The aim of this study was to fractionate a procyanidin-rich hawthorn bark extract using low-pressure liquid chromatography and to determine the compound profile and antioxidant activity of the obtained fractions. We identified and quantified the phenolics of four fractions (I–IV) separated on a Toyopearl HW-40S column with methanol as the mobile phase, using HPLC-DAD and LC-ESI-MS techniques. The antioxidant activity was determined to comprise ABTS^•+^ and DPPH^•^ scavenging activity, ferric-reducing antioxidant power (FRAP), and inhibition of β-carotene-linoleic acid emulsion oxidation. Characteristic data were subjected to principal component analysis (PCA). Fraction I contained mainly (−)-epicatechin (741.3 mg/g) and a lower amount of flavones and quercetin derivatives (100.7 mg/g). Fraction II was almost pure procyanidin B2, which accounted for 88.8% of the total phenolics. The subsequent fractions were rich in B-type procyanidin dimers, trimers, and tetramers. FRAP and antiradical activity against ABTS^•+^ and DPPH^•^ of the fraction containing low-molecular weight phenolics was lower than those of the fractions with procyanidin oligomers. The antioxidant activity of fractions II–IV ranged from 8.95 to 9.28 and from 6.45 to 6.71 mmol TE/g in the ABTS and DPPH assays, respectively. Their FRAP was in the range of 17.67–21.06 mmol Fe^2+^/g. According to PCA, the procyanidin dimers of fractions II and III were associated with antioxidant activity in these assays. In turn, the procyanidins with the highest degree of polymerization (trimers and tetramers) present in fraction IV were related to the antioxidant activity measured in the β-carotene-linoleic acid emulsion system. Overall, the separation of purified hawthorn bark extract using low-pressure Toyopearl HW-40S column chromatography resulted in a fraction rich in procyanidin B2, as well as fractions containing procyanidins with an increasing degree of polymerization, all with high levels of antioxidant activity under various conditions and the potential for future applications in food, pharmaceuticals, and cosmetics products.

## 1. Introduction

Common hawthorn (*Crataegus monogyna* Jacq., synonym *Crataegus oxyacantha* L. var. *praecox* hort. ex Loudon) is a plant belonging to the *Crataegus* L. genus in the Rosaceae family [1]. Its semi-evergreen shrubs or low trees (up to 15 m high) with thorny branches and small pome fruits are native to Europe, North Africa, Western Asia, and the Caucasus [1,2]. Hawthorn has long been considered a plant with therapeutic properties, and its fruit, leaves, and flowers have been traditionally used as a cardiotonic agent due to its antiatherogenic and blood pressure-lowering properties [2]. This herb has also been used as an astringent, antispasmodic, and diuretic. Modern in vitro and in vivo studies have confirmed the health-promoting properties of *C. monogyna* [2,3]. In addition to its protective effects on the cardiovascular system [4], hawthorn has been shown to have hepatoprotective, gastroprotective, antihypertensive, and anticancer properties [5,6,7]. It was also effective in the prevention on diabetes and neurological diseases [8,9,10]. These protective effects result from, among others, antioxidant [9], anti-inflammatory [11], immunomodulatory [12], and antimicrobial [6] activities of hawthorn secondary metabolites. Fruits, leaves, and flowers are rich sources of phenolic compounds, including phenolic acids, flavonoids, and proanthocyanidins, as well as terpenoids and organic acids [11,13,14,15]. Other morphological parts of hawthorn, such as seeds and bark, although less studied and less well understood, have also demonstrated different bioactivities due to their phytochemical composition [16,17,18]. Hawthorn bark may even be more attractive than the commonly used fruits and leaves; for example, Renda et al. [19] reported that hawthorn stem bark had a higher total phenolic content than the leaves and fruits. Furthermore, Włoch et al. [20], analyzing extracts of the bark and leaves, found higher free radical scavenging activity in the former, which was also more effective in protecting erythrocyte membrane lipids against oxidation.

The main phytochemicals of hawthorn bark are (−)-epicatechin and oligomeric procyanidins [20,21,22]. Procyanidins, or more broadly, proanthocyanidins (condensed tannins), are known for their diverse bioactivity, in particular their strong antioxidant properties [23,24,25]. These oligomers or polymers of flavan-3-ols, with multiple hydroxyl groups on aromatic rings in their structure, can easily scavenge free radicals, which determines the high antiradical potential of this group of phenolics [18,26]. Moreover, proanthocyanidins can interact with proteins through hydrophobic interactions or hydrogen bonds [25] and have the ability to chelate metal ions [27,28]. Both of these capabilities also enable proanthocyanidins to act as antioxidants. Forming complexes with enzymes involved in oxidation, proanthocyanidins inhibit their activity, while complexing reduced forms of transition metal ions including Fe^2+^ and Cu^+^ disrupts the oxidation reactions, in which these ions act as catalysts, e.g., the Fenton reaction. It has been shown that procyanidins isolated from hawthorn bark inhibited linoleic acid oxidation in a way comparable to Trolox and butylated hydroxytoluene (BHT) [29], and preparation from bark, rich in procyanidins, was an effective antioxidant after addition to food products like butter or bread [30,31]. The use of hawthorn bark as a functional food or cosmetic additive seems promising, especially since its aqueous and hydroalcoholic extracts did not show cytotoxic effects in a mouse model study, and genotoxic effects were only observed when very high doses (2000 mg/kg) were used [32].

The antioxidant activity of proanthocyanidins depends primarily on their degree of polymerization, which is correlated with molecular weight [24,33,34]. Furthermore, the flavan-3-ols skeleton, which determines the number and position of –OH groups in the molecule, and the type of bonds between monomer units (interflavan bond C4 → C8 and/or C4 → C6 for the B-type linkage and interflavan bond C4 → C8 and an additional ether bond between C2 and C7 for the A-type linkage) are important in this respect [24,35,36]. Therefore, structural analysis of proanthocyanidins of plant materials, and the isolation of compounds with the highest activity is crucial for their application. It is important to study individual plants, because both the plant-specific proanthocyanin composition and the matrix from which proanthocyanins are extracted can significantly determine the separability of these compounds and thus the available bioactivity. Although proanthocyanin-rich fractions with antioxidant potential have been obtained from many plants [24,34,35], hawthorn bark is poorly understood in this regard.

Chromatographic techniques are most commonly used for purifying proanthocyanidins from crude extracts and for their further fractionation and isolation [37,38]. Among them, low-pressure liquid chromatography on a Sephadex LH-20 column allows for both the separation of low-molecular-weight phenolics from proanthocyanidins [18,28] and the fractionation of the proanthocyanidins themselves based on their physicochemical properties, such as molecular weight or hydrophobicity [39,40]. Another commonly used stationary phase for fractionating this group of compounds is a Toyopearl HW resin [37]. Methanol or mixtures of acetone and water effectively eluted proanthocyanidins from different plant extracts loaded onto a Toyopearl HW column, depending on the degree of polymerization [41,42,43]. Based on this background, our studies aimed to fractionate a procyanidin-rich extract from hawthorn bark using a Toyopearl HW-40S column and methanol as an eluent to obtain fractions containing compounds with different structures, and after determining the antioxidant activity of these fractions in vitro in several assays, to find a relationship between the structure of hawthorn bark procyanidins and antioxidant activity.

## 2. Results and Discussion

### 2.1. Fractionation of Purified Hawthorn Bark Extract and Initial Fraction Characterization

**Figure 1:**
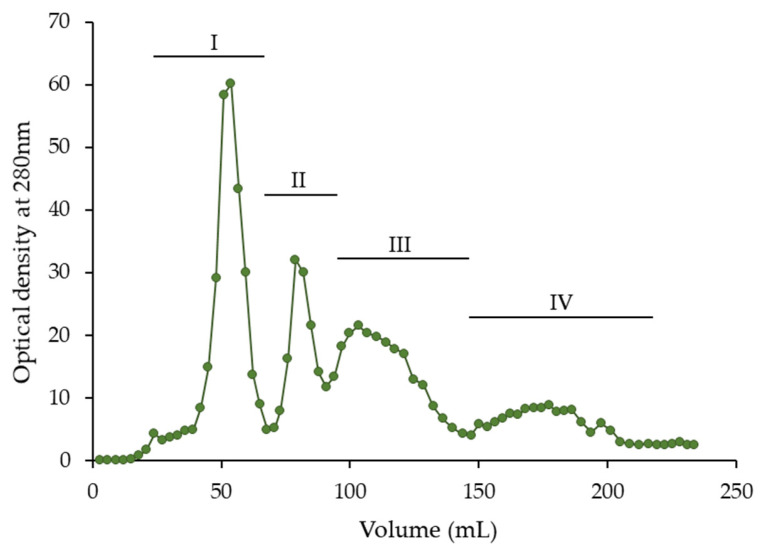
Separation of purified hawthorn bark extract using low-pressure Toyopearl HW-40S column chromatography.

Aqueous acetone (50–80%, *v*/*v*) is commonly used to extract plant materials high in proanthocyanidins [18,28,41] and is more effective than other aqueous organic solvents including aqueous ethanol or pure acetone and water [37]. In our study, the bark of young hawthorn twigs was extracted with 80% (*v*/*v*) acetone, and the crude extract was purified from ballast compounds, as proposed in previous studies [16,21,44], to concentrate phenolics and obtain an extract rich in flavan-3-ols and their oligomers. Next, the purified extract was separated by low-pressure liquid chromatography on a Toyopearl HW-40S column to obtain fractions containing various types of phenolic compounds. The chromatogram of this separation, with absorbance monitoring at 280 nm, is shown in Figure 1. Four peaks are visible in the chromatogram (I–IV). The eluent corresponding to each peak was fractionated. After evaporation of the methanol, dried fractions I–IV were obtained, the mass of which constituted 10.3–24.6% of the mass of purified extract (Table 1).

The purified hawthorn bark extract and fractions were initially characterized by determination of total phenolic content (TPC) and condensed tannin (CT) content. The results of these analyses are shown in Table 1. The TPC of extract (801.2 mg gallic acid equivalent (GAE)/g) was significantly lower (*p* < 0.05) than those of fractions II–IV and similar (*p* ≥ 0.05) to that of fraction I. Among fractions, the highest TPC was found in fraction IV (896.5 mg GAE/g), followed by fraction II (862.8 mg GAE/g). The TPC of the extract was high compared to literature data for crude and purified extracts from bark and other parts of hawthorn [6,9,11,13,19]. Turnalar Ülger et al. [11] reported that the TPC of crude hydroethanolic (70%, *v*/*v*) extracts from flower-bearing branches of different *Crataegus* species ranged from 239.09 to 368.94 mg GAE/g, with the value for the *C. monoygna* being 301.77 mg GAE/g. Leaf and flower extracts of this species contained lower amounts of total phenolic compounds—169.66 and 116.24 mg GAE/g, respectively. In turn, crude extracts from hawthorn stem bark obtained using acidified and non-acidified methanol and ethanol all had TPCs above 100 mg GAE/g, up to 132.26 g/g [19]. Goudjil et al. [13] extracted a mixture of hawthorn leaves, fruits, and flowers with 80% (*v*/*v*) methanol and then re-extracted crude extract with various solvents. They found that the *n*-butanol fraction had a TPC of 330 mg GAE/g. The high TPC of the purified extract in our study indicates effective removal of ballast compounds such as chlorophylls, lipids, and carbohydrates, which are typically extracted from matrix together with phenolics with aqueous acetone [38]. Such purification also contributed to the relatively high TPC of the fractions, which the phenolic compounds did not share with other compounds originating from the matrix.

The CT content in the extract and its fractions ranged from 1.58 to 2.66 A_500_/mg (Table 1). The highest CT content was found in fractions II and IV, followed by fraction III. In contrast to TPC, the extract had a significantly (*p* < 0.05) higher CT content than fraction I. Therefore, it can be assumed that phenolic compounds other than condensed tannins were eluted at the beginning of the separation and enhanced fraction I. The CT content of purified hawthorn extract was high compared to literature data, e.g., for tannin fractions separated from almonds, red lentil, and buckwheat (0.207–1.036 A_500_/mg) [28].

### 2.2. Phenolic Profile of Purified Hawthorn Bark Extract and Its Fractions

**Figure 2:**
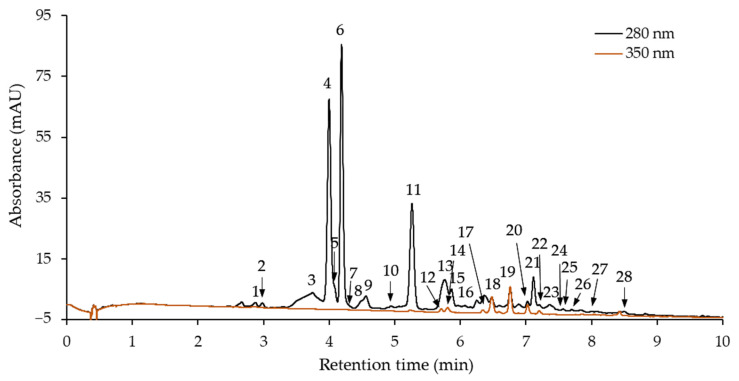
High-performance liquid chromatography with diode array detection (HPLC-DAD) chromatogram of purified hawthorn bark extract.

Phenolic compounds of purified hawthorn bark extract and its fractions were identified and quantified using high-performance liquid chromatography with diode array detection (HPLC-DAD) and liquid chromatography coupled to electrospray ionization mass spectrometry (LC-ESI-MS). The HPLC-DAD chromatogram of the extract is shown in Figure 2. Twenty-eight peaks corresponding to phenolic compounds were identified. Each of these compounds was also identified in at least one fraction obtained after separation on a Toyopearl HW-40S column. Peaks numbered 5, 7, 16, 24, 26, and 27 are barely visible, because they correspond to compounds present in trace amounts in extract. Retention times and UV absorption maxima (λ_max_) from DAD are listed in Table 2, along with the mass spectrometric data including negative molecular and fragment ions of eluted compound.

Compounds **2**, **4**, and **6** were identified as (+)-catechin, procyanidin B2, and (−)-epicatechin, respectively, by comparing their retention times and UV and mass spectra with those of the commercial standards. In addition to procyanidin B2, five other B-type procyanidin dimers were tentatively identified, namely compounds **1**, **3**, **8**, **16**, and **21**. All of them were characterized by a λ_max_ at 278–280 nm and an [M − H]^−^ ion at *m*/*z* 577, indicating B-type procyanidin dimers [45]. Furthermore, a characteristic ion was detected at *m*/*z* 289 [M − H − 288]^−^. It was formed from the base (+)-catechin or (−)-epicatechin unit of the dimer after cleavage of the interflavan bond via the quinone methide (QM) mechanism [45,46]. Another fragment ion of mass spectra of compounds **1** and **8** at *m*/*z* 425 [M − H − 152]^−^ resulted from the neutral loss of the B ring with two hydroxyl groups from the top flavan-3-ol unit ((+)-catechin or (−)-epicatechin) via a retro-Diels Alder (RDA) reaction of the C ring [45,46]. Among flavan-3-ols and their dimers, (−)-epicatechin and procyanidins B2, B4, and B5 were reported in hawthorn bark [21,29,44]. Furthermore, Wyspiańska et al. [16] classified three other compounds of hawthorn bark preparation as B-type procyanidin dimers, and Włoch et al. [20] identified a total of three B-type procyanidin dimers in extract. (+)-Catechin was not detected in any of the cited studies.

Compounds **5**, **7**, **9**–**11**, **15**, **23**, and **17** shared common characteristics, e.g., λ_max_ at 278–280 nm, a molecular ion at *m*/*z* 865, and the fragment ions at *m*/*z* 577 and 289 (Table 2). An additional fragment ion at *m*/*z* 575 was detected in the mass spectra of compounds **9**, **11**, and **23**. All of them were tentatively identified as B-type procyanidin trimers. The ions at *m*/*z* 865 [M − H]^−^ and at *m*/*z* 577 [M − H − 288]^−^ are indicative of B-type trimers consisting exclusively of (+)-catechin and/or (−)-epicatechin [46]. The ion at *m*/*z* 577 derived from QM cleavage of the interflavan bonds between the top and the middle units of the trimer, and the ions at *m*/*z* 575 and 289 were the products of QM cleavage of the interflavan bond between the middle and the base units [45,47]. In turn, in mass spectra of compounds **13**, **26**, and **28**, an [M − H]^−^ ion at *m*/*z* 1153 and a series of daughter ions at *m*/*z* 865, 577, and 289 were detected (Table 2). The [M − H − n × 288]^−^ ions correspond to the fragmentation of procyanidins with the B-type linkage [47]. Therefore, these compounds were tentatively identified as B-type procyanidin tetramers. Both trimers and tetramers with the B-type linkage have been previously identified in hawthorn bark [16,20].

The molecular ion of A-type procyanidin dimers is 2 Da smaller than that of B-type procyanidin dimers [45,47]. There is also a 2 Da difference between the [M − H]^−^ ions of B-type procyanidin trimers and procyanidin trimers with one A-type linkage. On this basis, compound **24** ([M − H]^−^ ion at *m*/*z* 863) and compound **25** ([M − H]^−^ ion at *m*/*z* 575) were tentatively identified as A-type procyanidin trimer and dimer, respectively. The fragment ion at *m*/*z* 575 in the mass spectrum of compound **24**, formed after QM cleavage of the interflavan bond, indicated that the A-type linkage was between the middle and base units of the trimer [45].

In addition to flavan-3-ols and their oligomers, four compounds classified as *C*-glycosylated flavones (**12**, **14**, **17**, and **19**) and three *O*-glycosylated flavonols (compounds **18**, **20**, **22**) were identified in purified hawthorn bark extract and its fractions (Table 2). Two luteolin *C*-glucosides were recognized based on their λ_max_ at 268 and 348 nm and an [M − H]^−^ ion at *m*/*z* 447 [48,49]. They were than identified as isoorientin (compound **12**) and orientin (compound **14**) by comparing their retention times with those of commercial standards. The [M − H]^−^ ion at *m*/*z* 431, the second λ_max_ at a shorter wavelength (336–338 nm), and the characteristic fragment ions were the basis for classifying compounds **17** and **19** as apigenin *C*-glucosides [20,48,49]. Analysis of the standards allowed for their further identification as vitexin and isovitexin, respectively. In turn, a fragment ion at *m*/*z* 301 was detected in the mass spectra of compounds **18**, **20**, and **22** (Table 2). Based on this ion, corresponding to the quercetin moiety, and the [M − H]^−^ ion at *m*/*z* 463, after comparison with the data for standards, compounds **20** and **22** were identified as quercetin 3-*O*-galactoside and quercetin 3-*O*-glucoside, respectively. The structure of compound **22** could not be determined and was considered a quercetin derivative. The presence of apigenin and luteolin *C*-glucosides and quercetin *O*-glycosides in hawthorn bark is consistent with findings in the literature [20,22].

The quantitative profile of compounds identified in the purified hawthorn bark extract and its fractions is presented in Table 3. In extract, flavan-3-ols, procyanidin dimers, procyanidin trimers, and procyanidin tetramers constituted 28.9%, 40.8%, 20.2%, and 6.5% of total phenolics, respectively. Flavones and flavonols (other flavonoids) accounted for only 3.5% of total phenolics. The total content of flavan-3-ols and their oligomers was 715.9 mg/g extract, and this value fell within the range of 576.55–761.9 mg/g reported in the literature for hawthorn bark extracts and preparations [16,20,44]. In turn, total content of flavones and flavonols was slightly higher than the 14.96 mg/g found for the aqueous extract of hawthorn bark purified on AP 400 resin [20].

Almost all flavan-3-ols of the extract were distributed in fraction I, where their content was 751.6 mg/g (85.6% of the total phenolics) (Table 3). Flavan-3-ols were not detected in fractions III and IV, and their level in fraction II was negligible (2.5 mg/g). Procyanidin dimers were determined in extract and fractions I–III, but their share in fraction I was very low (2.8% of the total phenolics). Most of them were eluted from extract into fraction II, and accounted for 98.7% of the total phenolics in this fraction. In fraction III, the procyanidin dimer content was 439.7 mg/g (52.8% of the total phenolics). The content of procyanidin trimers was the highest in fraction IV (502.0 mg/g), although they were also found in significant amounts in fraction III (393.3 mg/g). The contribution of this group of compounds in the total phenolics of fraction III and IV was 47.2% and 59.2%, respectively. Trimers were not identified in fraction I, and their quantity in fraction II was negligible. Procyanidin tetramers were found only in the extract and fraction IV. In the latter, their content was 345.5 mg/g. The other identified phenolic compounds were almost entirely separated into fraction I. In fraction II, they constituted only 0.6% of the total phenolics, and they were not detected in the fractions III and IV. This finding was in agreement with the TPC and CT content and confirmed our assumption that compounds other than condensed tannins were eluted into fraction I.

As for individual phenolic compounds, (−)-epicatechin, procyanidin B2, and B-type procyanidin trimer(5) were the main compounds of the extract, with contents of 211.9, 187.5, and 100.1 mg/g, respectively. (−)-Epicatechin constituted 98.7% of the total flaval-3-ols. The level of (+)-catechin was negligible. Due to the high content in the extract and the fact that flavan-3-ols were separated into fraction I, (−)-epicatechin was the dominant compound of this fraction (741.3 mg/g). The next in decreasing order of content were quercetin 3-*O*-galactoside and orientin, for which up to 18- and 29-fold lower values were obtained, respectively. Procyanidin B2 was the main compound of fraction II (770.6 mg/g). The content of the two other B-type procyanidin dimers, designated as 1 and 2, was only 53.8 and 17.0 mg/g fraction, respectively. The remaining compounds of fraction II were quantified at levels lower than 10 mg/g. The B-type procyanidin dimer(2) was mainly distributed in fraction III, where its content was 278.6 mg/g, but the content of B-type procyanidin trimer(5) was even higher in this fraction (332.0 mg/g). The latter compound was also eluted into fraction IV and constituted 21.9% of the total phenolics. However, the dominant compound of fraction IV was B-type procyanidin tetramer(1), with a content of 314.4 mg/g. This content was very high compared to the content of B-type procyanidin tetramer(1) in the extract. In the latter case, underestimation could have occurred due to interference between the peak corresponding to this compound and those of orientin and isoorientin, which were also present in the extract. A similar problem occurred during the quantification of B-type procyanidin trimer(1) in the extract, for which the corresponding peak overlapped with the peak from (−)-epicatechin. Nevertheless, the quantitative profiles of the main compounds were consistent with the literature data for hawthorn bark, which also showed the highest content of (−)-epicatechin, followed by procyanidin B2 [21,29]. Among other main oligomers, a trimer—procyanidin C1—and two dimers—procyanidins B2 and B4—were reported.

Toyopearl HW-40S resin is designed for size exclusion chromatography, and the separation of procyanidin oligomers with different degrees of polymerization was expected. Indeed, fraction I with (−)-epicatechin and other low-molecular-weight phenolic compounds and fraction II with procyanidin dimers were obtained using a Toyopearl HW-40S column and methanol as the mobile phase. The subsequent fractions contained procyanidins with increasingly higher molecular weight and degrees of polymerization (fraction III consisted of dimers and trimers, and fraction IV contained trimers and tetramers); however, a clear partition between dimers, trimers, and tetramers was not achieved. Such fractionation was shown in previous studies, in which oligomeric procyanidins of litchi pericarp and Granny Smith apple were separated [42,43]. Nevertheless, due to the numerous –OH groups in procyanidin’s structure, it can interact with the Toyopearl HW resin through hydrogen bonding and hydrophobic effects and interfere the separation based solely on molecular sieving [37]. Furthermore, Chen et al. [40] found that procyanidins with increasing molecular weight or degree of polymerization exhibited decreasing polarity, decreased ability to form hydrogen bonds, and enhanced hydrophobic interactions. These properties of procyanidins may suggest different strengths of the interfering interaction with Toyoperyl HW-40S resin and thus explain the incomplete separation of dimers from trimers and trimers from tetramers in our study.

### 2.3. Antioxidant Activity of Purified Hawthorn Bark Extract and Its Fractions

**Figure 3:**
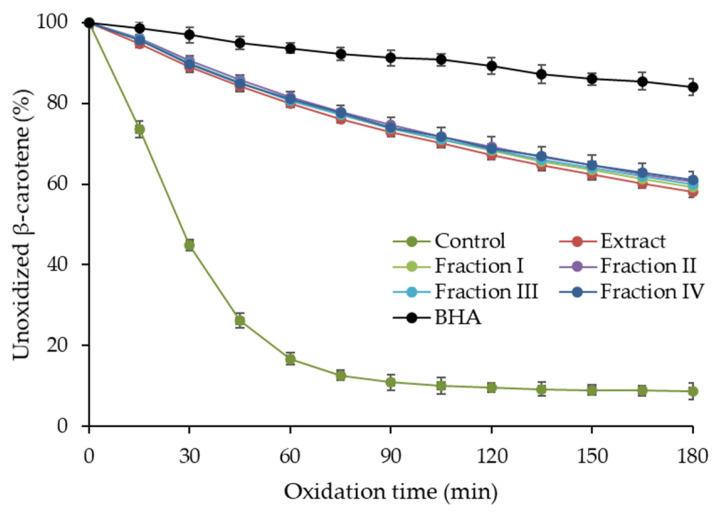
Antioxidant activity of purified hawthorn bark extract and its fractions separated on a Toyopearl HW-40S column in β-carotene-linoleic acid emulsion model. BHA, butylated hydroxyanisole.

Due to the diverse mechanism of action of phenolic compounds as antioxidants, several assays were carried out to investigate the antioxidant activity of purified hawthorn bark extract and its fractions. Antiradical activity against the 2,2′-azino-bis(3-ethylbenzothiazoline-6-sulfonic acid) radical cation (ABTS^•+^) and the 2,2-diphenyl-1-picrylhydrazyl radical (DPPH^•^), as well as ferric-reducing antioxidant power (FRAP) were examined. The results of these assays are presented in Table 4. Furthermore, the ability of the extract and fractions to the inhibit oxidation of the β-carotene-linoleic acid emulsion was analyzed, and the oxidation curves are shown in Figure 3.

The ABTS^•+^ scavenging activity of the extract was 8.48 mmol Trolox equivalent (TE)/g (Table 4). The values for the fractions ranged from 7.86 to 9.28 mmol TE/g and fraction I exhibited significantly lower (*p* < 0.05) antiradical activity against ABTS^•+^ than fractions II–IV. The trend of variation in DPPH^•^ scavenging activity of the fractions was similar, with a significantly lower value (*p* < 0.05) for fraction I (4.95 mmol TE/g) compared to the others (6.45–6.71 mmol TE/g). The DPPH^•^ scavenging activity of the extract was significantly lower than that of fractions II–IV, but higher than that of fraction I. The extract and fractions were more diverse in terms of FRAP, which was in the range of 13.91–21.06 mmol Fe^2+^/g and increase significantly in the following order: fraction I < fraction III = fraction IV = extract < fraction II. In turn, β-carotene-linoleic acid emulsion oxidation was inhibited by the extract and all fractions similarly (Figure 3) and no significant differences (*p* ≥ 0.05) in inhibition were found between them after 180 min of the process. All samples showed a lower ability to inhibit emulsion oxidation than the synthetic antioxidant—butylated hydroxyanisole (BHA). However, considering that BHA and samples were used at the same concentration, it can be concluded that the activity of the extract and the fraction was relatively high. Comparing with literature data, the results of the ABTS assays fell within the range of 6.10–12.9 mmol TE/g reported for purified hawthorn bark extracts, while a lower DPPH^•^ scavenging activity was noted (1069 µmol TE/g) [16,20]. As expected, the antioxidant activity of the purified extract and fractions was higher than that reported for the crude hawthorn extracts. For example, the antiradical activity against ABTS^•+^ and DPPH^•^ of hydroalcoholic extracts from flowering branches of different *Crataegus* species ranged from 319.29 to 466.59 mg TE/g and 380.78 to 457.27 mg TE/g, respectively [11]. Leaf and fruit extracts were characterized by even lower activity in these assays.

Fraction I, containing flavan-3-ols and other flavonoids, showed lower antioxidant activity in most assays (ABTS, DPPH, and FRAP) than fractions II–IV, which were rich in procyanidin oligomers. This may be due to the fact that (−)-epicatechin, the main compound of fraction I, is a worse free radical scavenger than its oligomers [26]. Another reason may be the presence of quercetin glycosides in fraction I. Lu and Yeap Foo [26] found that quercetin glucoside, galactoside, rhamnoside, arabinoside, and xyloside have lower O_2_^•−^ scavenging activity than (−)-epicatechin and its dimer, trimer, and tetramer. Among the other flavonoids of fraction I, vitexin and isovitexin contributed rather insignificantly to its antioxidant activity. This is because the ABTS^•+^ and DPPH^•^ scavenging activity and FRAP of apigenin *C*-glucosides are reportedly low; several times lower than those of luteolin *C*-glucosides [48]. In turn, the lack of differences between fraction I and the others in inhibiting β-carotene-linoleic acid emulsion oxidation can be explained by the lipophilic reaction environment, in which monomers and oligomers may act as antioxidant differently as in aqueous systems of ABTS, DPPH, and FRAP assays. Plumb et al. [36] found that polymerization of catechin into oligomers significantly reduced antiradical activity in the lipid system, while simultaneously increasing activity in the aqueous system of the ABTS assay, which is in agreement with our findings.

The antioxidant activity of fractions containing procyanidin oligomers with increasing degrees of polymerization (fraction II with dimer, fraction III with dimers–trimers, fraction IV with trimers–tetramers) did not differ significantly, except for FRAP. Although some studies have shown a positive correlation between antioxidant activity and the degree of polymerization of proanthocyanidin oligomers, this relationship may vary depending on the antioxidant assay system, the source of proanthocyanidins, and their type [24]. Furthermore, the correlations were observed up to a certain degree of polymerization, above which the oligomers showed a decrease in antioxidant activity. For example, Zhou et al. [34] obtained proanthocyanidin fractions from mangosteen pericarp and reported that fractions with a mean degree of polymerization ranging from 2.71 to 9.27 were characterized by increasing DPPH^•^ scavenging activity and FRAP, but all showed similar results in the ABTS assay. Lu and Yeap Foo [26] found that (−)-epicatechin oligomers isolated from Gala apple pomace exhibited increasing O_2_^•−^ and DPPH^•^ scavenging activity and antioxidant activity in the β-carotene and linoleic acid system up to trimers; tetramers showed similar activity to trimers. A-type procyanidin oligomers from litchi pericarp were also characterized by increasing antiradical activity against DPPH^•^ and ^•^OH up to trimers [33]. In the case of cinchonain and B-type trimer, tetramer, and pentamer isolated from chokeberry, a positive correlation was found between the degree of polymerization and antiradical activity against ABTS^•+^, but in a cell-based assay system, only trimer and tetramer showed antioxidant activity [35]. In contrast, Chai et al. [39], who fractionated proanthocyanidins from *Ficus altissima* bark, found that the mean degree of polymerization was negatively correlated with the antioxidant activity of the obtained fractions in ABTS and DPPH assays.

### 2.4. Overall Estimation of Results Using Principal Component Analysis

**Figure 4:**
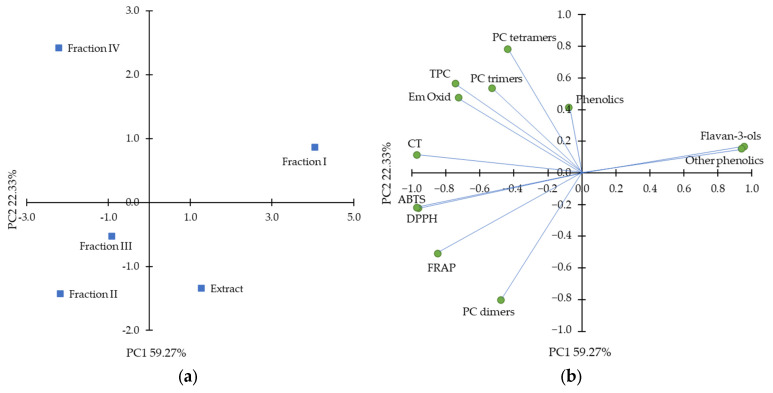
Principal component analysis plots with (**a**) distribution of objects and (**b**) distribution of the variables, including total phenolic content (TCA), condensed tannin (CT) content, ferric-reducing antioxidant power (FRAP), ABTS^•+^ scavenging activity (ABTS), DPPH^•^ scavenging activity (DPPH), inhibition of β-carotene-linoleic acid emulsion oxidation after 180 min (Em Oxid) and sum of different class of phenolics from high-performance liquid chromatography analysis.

A data set of composition and antioxidant activity of purified hawthorn bark extract and its fractions was subjected to principal component analysis (PCA) to uncover any patterns between the characteristics. The results are shown in Figure 4. The two first principal components (PC1 and PC2) explained 81.60% of the total variance. Among the variables, ABTS, DPPH, and FRAP assays were clustered. These assays measuring antioxidant activity in a polar environment were separated along the PC2 from antioxidant activity in a β-carotene-linoleic acid emulsion system, which indicates the importance of the assay conditions. As shown in the object distribution plot, fraction I was clearly discriminated from the other fractions and the extract. Furthermore, along PC2, fraction IV was distinguished from fractions II and III. Fraction I, as expected, was associated with total flavan-3-ols and other low-molecular-weight compounds, but these variables did not correlate positively with antioxidant activity in any antioxidant assay. Fractions II and III were clustered by variables such as antiradical activity against ABTS^•+^ and DPPH^•^, FRAP, and total procyanidin dimers. Interestingly, PCA showed a clear relationship between fraction IV with procyanidin trimers and tetramers and antioxidant activity measured as the ability to inhibit oxidation of β-carotene-linoleic acid emulsion, suggesting that procyanidins with the highest degree of polymerization were more active under lipophilic condition than compounds of fractions II and III.

## 3. Materials and Methods

### 3.1. Material, Chemicals, and Reagents

The experiment was carried out using the bark of young twigs from trees of common hawthorn (*Crataegus monogyna* Jacq.) growing in a local garden.

Folin–Ciocalteu’s reagent, gallic acid, vanillin, β-carotene, Tween 40, linoleic acid, BHA, 2,2′-azino-bis(3-ethylbenzothiazoline-6-sulfonic acid) diammonium salt (ABTS), 2,2-diphenyl-1-picrylhydrazyl (DPPH) radical, 2,4,6-tri(2-pyridyl)-*s*-triazine (TPTZ), Trolox, and the gradient grade solvents for HPLC were purchased from Sigma-Aldrich (St. Louis, MO, USA). HPLC standards including (+)-catechin, (−)-epicatechin, procyanidin B2, quercetin 3-*O*-glucoside, quercetin 3-*O*-galactoside, orientin, isoorientin, vitexin, and isovitexin were acquired from Extrasynthese (Genay, France). The purity of procyanidin B2 and quercetin 3-*O*-galactoside was ≥90% and ≥98%, respectively. All other HPLC standards were ≥99% pure. The solvents and reagents, if not otherwise specified, were acquired from Avantor Performance Materials Poland S.A. (Gliwice, Poland). All of them were analytical grade.

### 3.2. Extraction of Hawthorn Bark and Purification of Crude Extract

The hawthorn bark was cut into fine particles with a knife and extracted using the method of Oszmiański and Bourzeix [21]. Briefly, approximately 150 g of bark was poured into 1.5 L of 80% (*v*/*v*) aqueous acetone and sonicated for 15 min at 20 °C. The mixture was saturated with nitrogen and left at −22 °C for 24 h. After filtration, crude extract was purified according to the procedure described by Wyspiańska et al. [16]. For this purpose, chloroform was added to the crude extract (1:2, *v*/*v*) to remove non-polar compounds. After shaking and separation, the chloroform phase was discarded, and the acetone was evaporated from the second phase using a vacuum evaporator (Rotavapor R-200, Büchi Labortechnik, Flawil, Switzerland). The aqueous residue was then re-extracted with ethyl acetate (10:1, *v*/*v*). To remove any remaining water, the ethyl acetate phase was filtered through anhydrous Na_22_SO_44_. In the final step, *n*-hexane was used to precipitate the solids, which were separated by centrifugation. The resulting purified extract was dried and ground to a uniform powder.

### 3.3. Fractionation of Purified Hawthorn Bark Extract

The purified hawthorn bark extract was fractionated by low-pressure liquid chromatography according to the method of Oszmiański and Bourzeix [21] with some modifications. A 2 cm internal diameter glass column was packed with Toyopearl HW-40S resin with a particle size of 30 µm (Tosoh Bioscience, Tokyo, Japan) to a height of 30 cm, then washed with a mixture of acetone and water (4:1, *v*/*v*) and then with methanol. A 300 mg portion of the purified extract dissolved in 2 mL of methanol was loaded onto a column, and elution was initiated with methanol as the mobile phase with a flow rate of 0.4 mL/min. The eluate void volume (30 mL) was discarded, and then 4 mL aliquots were collected using a RediFrac fraction collector (Amersham Pharmacia Biotech, Amersham, UK). The absorbance of each aliquot was measured at 280 nm using a DU-7500 diode array spectrophotometer (Beckman Instruments, Fullerton, CA, USA). Aliquots were combined into four fractions based on chromatogram plots, and then dried by methanol evaporation using a vacuum evaporator, and weighted. The fraction yield was calculated as the ratio of the fraction weight to the weight of the extract used for separation and expressed as a percentage. The separation was performed in triplicate.

### 3.4. Determination of Total Phenolic Content

The total phenolic content of the purified hawthorn bark extract and its fractions was determined by reaction with Folin–Ciocalteu’s reagent [50]. Briefly, the extract or fraction was dissolved in methanol to obtain solution with concentration of 0.1 mg/mL. To 0.1 mL of this solution was added 0.1 mL of twice-diluted Folin–Ciocalteu’s reagent and 0.2 mL of saturated Na_2_CO_3_ solution. The reaction mixture was then adjusted to 2 mL with water and, after 25 min of color development, centrifuged for 5 min at 5000× *g* using a MiniSpin plus centrifuge (Eppendorf, Hamburg, Germany). A DU-7500 spectrophotometer set at 725 nm was used to measure the absorbance of the supernatant. Results were calculated from a standard curve (y = 5.65x − 0.01, correlation coefficient, r = 0.999) plotted for gallic acid solutions in the concentration range of 0.02–0.10 mg/mL, and expressed as mg of GAE per g of extract or fraction.

### 3.5. Determination of Condensed Tannin Content

To determine the content of condensed tannins, methanolic solutions of purified hawthorn bark extract and its fractions were reacted with vanillin/HCl reagent, which was prepared by dissolving 0.5 g of vanillin in 100 mL of 4% (*v*/*v*) HCl in methanol [51]. Sample solutions at a concentration of 0.1 mg/mL were mixed with the vanillin/HCl reagent in a 1:5 (*v*/*v*) ratio. Color was developed in the dark for 20 min, and absorbance was measured at 500 nm. In parallel, control samples were prepared with 4% (*v*/*v*) HCl in methanol instead of the vanillin/HCl reagent. Results were expressed as absorbance per mg of extract or fraction (A_500_/mg).

### 3.6. Identification and Quantification of Phenolic Compounds

The phenolic profile of purified hawthorn bark extract and its fractions was analyzed using the Shimadzu HPLC system equipped with LC-30AD pumps, a SIL-30AC autosampler, an SPD-M30A diode array detector, and a CBM-20A controller (Shimadzu, Kyoto, Japan). A Kinetex C18 column (75 × 3 mm, 2.6 µm particle size, 100 Å pore size, Phenomenex, Torrance, CA, USA) was used to separate samples dissolved in methanol (1 mg/mL), filtered through a nylon membrane (0.22 µm), and injected into the column at a volume of 2.5 µL. The column temperature was maintained at 20 °C. Elution was performed in a binary gradient system of solvent A (acetonitrile–water–trifluoroacetic acid at a ratio of 5:95:0.1, *v*/*v*/*v*) and solvent B (acetonitrile–trifluoroacetic acid at a ratio of 100:0.1, *v*/*v*) with 20% B from 0 to 10 min, increasing B to 85% from 10 to 13 min, and decreasing B to 0% from 13 to 14 min. The mobile phase flow rate was 1 mL/min. The spectra of the analytes were monitored in the wavelength range of 200–400 nm. For quantification of flavan-3-ols and their oligomers, the absorbance was measured at 280 nm, and for the remaining flavonoids, at 350 nm. Results were calculated using calibration curves (r ≥ 0.998) of appropriate standards, including (+)-catechin, (−)-epicatechin, procyanidin B2, quercetin 3-*O*-glucoside, quercetin 3-*O*-galactoside, orientin, isoorientin, vitexin, isovitexin, or structurally related compounds. The content of individual phenolic compounds was expressed as mg per g of extract or fraction.

Mass spectra of the eluted compounds were obtained using an HP 1100 Series liquid chromatograph with a mass selective detector, LC/MSD (Hewlett Packard, Palo Alto, CA, USA). The system was equipped with an atmospheric pressure ionization (API) source. MS measurements were recorded using electrospray ionization in the negative ion mode. N_2_ was used as the nebulizing gas at 275 kPa and as drying gas with a flow rate of 10 L/min at 340 °C. Voltage at the capillary entrance was 4000 V, and variable fragmentation voltage was 100 V (*m*/*z* 200–1000) and 250 V (*m*/*z* 1000–2500). Mass spectra were recorded from *m*/*z* 100 to *m*/*z* 2500.

### 3.7. Determination of ABTS Radical Cation Scavenging Activity

The ABTS^•+^ decolorization assay was performed according to the original procedure of Re et al. [52]. Briefly, ABTS^•+^ was generated by a reaction of 192 mg of ABTS and 33 mg of K_2_S_2_O_8_ in 50 mL of water. After standing overnight, the solution was diluted with methanol to a final absorbance of 0.70 ± 0.02 at 734 nm. The reaction of 20 μL of methanolic solutions of purified hawthorn bark extract and fractions (concentration 0.1 mg/mL) with 2 mL of ABTS^•+^ solution proceeded at 30 °C for 6 min. Absorbance was measured at 734 nm. Results were calculated from a Trolox standard curve (y = 43.9x + 0.18, r = 0.999) prepared for solutions in the concentration range of 0.4–2.0 µmol/mL and expressed as mmol of TE per g of extract or fraction.

### 3.8. Determination of DPPH Radical Scavenging Activity

The DPPH^•^ scavenging activity of purified hawthorn bark extract and its fractions was determined by reacting 0.25 mL of 1 mM DPPH^•^ solution with 100 μL of 0.1 mg/ mL sample solution, both in methanol, after adding 2 mL of methanol [53]. The reaction was performed at 20 °C in the dark for 20 min, and then the absorbance at 517 nm was measured. The Trolox standard curve (y = −0.947x + 1.19, r = 0.998) for the concentration range of 0.12–1.20 µmol/mL was used to calculate the results, which were expressed as mmol of TE per g of extract or fraction.

### 3.9. Determination of Ferric-Reducing Antioxidant Power

FRAP of purified hawthorn bark extract and its fractions was determined according to the original procedure of Benzie and Strain [54]. A FRAP reagent consisting of one volume of 10 mM TPTZ (in 40 mM HCl), one volume of 20 mM FeCl_3_ × 6H_2_O, and ten volumes of 300 mM acetate buffer (pH 3.6) was mixed with the sample dissolved in methanol at a concentration of 0.1 mg/mL. The amounts of FRAP reagent and sample solution taken for the reaction were 2.25 mL and 75 μL, respectively, and the final volume was adjusted to 2.55 mL with water. The reaction was allowed to develop at 37 °C for 30 min, and absorbance was measured at 593 nm. Results were calculated using the calibration curve (y = 0.632x − 0.02, r = 0.996) for FeSO_4_ (concentration range 0.2–2.0 µmol/mL) and expressed as mmol of Fe^2+^ equivalent per g of extract or fraction.

### 3.10. Oxidation of β-Carotene-Linoleic Acid Emulsion

The ability of purified hawthorn bark extract and its fractions to inhibit oxidation of β-carotene-linoleic acid model emulsion was determined by the Miller method, according to the protocol described in our previous publication, which was adapted from the method used to carry out the reaction on a 96-well plate [55]. Briefly, an emulsion was prepared by dissolving 2 mg of β-carotene, 400 mg of Tween 40, and 40 μL of linoleic acid in 2 mL of chloroform. After evaporation of chloroform under nitrogen, 2 mL of methanol was added, followed by 50 mL of water. Aliquots of emulsion (250 μL) were mixed with 10 μL of sample solutions in methanol (1 mg/mL) or BHA (1 mg/mL) on a plate and reacted at 42 °C for 3 h. A control sample containing methanol instead of the antioxidant solution was also oxidized. Absorbance was measured at 15 min intervals at 470 nm using an Infinite M1000 microplate reader (Tecan, Männedorf, Switzerland). Results were expressed as percent of unoxidized β-carotene.

### 3.11. Statistical Analysis

Analyses were performed in three replicates and the results were expressed as mean and standard deviation. To determine the significance of differences between the purified hawthorn bark extract and each fraction, one-way analysis of variance with Tukey’s post hoc test was performed using GraphPad Prism software, version 6.04 (GraphPad Software, Boston, MA, USA). Differences were considered significant at *p* < 0.05. Moreover, data were subjected to PCA using Statistica 14.1.0.4 software (Cloud Software Group, Inc., Palo Alto, CA, USA).

## 4. Conclusions

Flavan-3-ols (mainly (−)-epicatechin), B-type procyanidin dimers (mainly procyanidin B2), and B-type procyanidin trimers and tetramers were found to be the main compounds of purified hawthorn bark extract. Compounds classified as *C*-glycosylated flavones and quercetin derivatives were also identified. The use of low-pressure Toyopearl HW-40S column chromatography with methanol as the mobile phase allowed for the separation of flavan-3-ols and other low-molecular-weight phenolic compounds of the extract from procyanidin oligomers, as well as very clear separation of a fraction containing almost only procyanidin B2. Although procyanidin oligomers with increasingly high degrees of polymerization were eluted into subsequent fractions (dimers and trimers in fraction III and trimers and tetramers in fraction IV), no fractions containing only procyanidin trimers or tetramers were obtained.

The antioxidant activity of hawthorn bark extract and its fractions was dependent on the assay used, but in general, procyanidin oligomers guaranteed higher antioxidant activity of fractions II–IV compared to fraction I, which contained mainly low-molecular-weight phenolics, including (−)-epicatechin and other flavonoids. Differences in antioxidant activity between fractions containing procyanidin dimers, trimers, and tetramers were insignificant or minor. Nevertheless, PCA revealed an association between procyanidins with the highest degree of polymerization (trimers and tetramers) and antioxidant activity measured under lipophilic conditions of β-carotene-linoleic acid emulsion oxidation.

Overall, hawthorn bark extract can easily yield almost pure procyanidin B2, as well as fractions containing B-type procyanidin dimers–trimers and B-type procyanidin trimers–tetramers, all with high antioxidant activity and potential for future applications in the food, pharmaceutical, and cosmetics industries. However, such applications require cell-based and in vivo studies of hawthorn bark fractions to confirm the significance of the in vitro findings. Further in-depth studies of the hawthorn bark procyanidin structures tentatively identified in this study are also necessary.

## Figures and Tables

**Table 1:** Total phenolic content (TPC) and condensed tannin (CT) content of purified hawthorn bark extract and its fractions separated on a Toyopearl HW-40S column, as well as fraction yield.

Extract/Fraction	Fraction Yield (%)	TPC (mg GAE/g)	CT Content (A_500_/mg)
Extract	–	801.2 ± 11.4 ^d^	1.88 ± 0.08 ^c^
Fraction I	24.6 ± 1.5 ^a^	816.3 ± 6.5 ^cd^	1.58 ± 0.03 ^d^
Fraction II	11.8 ± 0.9 ^c^	862.8 ± 8.1 ^b^	2.65 ± 0.11 ^a^
Fraction III	19.0 ± 1.3 ^b^	826.3 ± 7.4 ^c^	2.21 ± 0.02 ^b^
Fraction IV	10.3 ± 1.7 ^c^	896.5 ± 11.1 ^a^	2.66 ± 0.04 ^a^

Results are presented as mean ± standard deviation (*n* = 3). Different superscript letters (^a–d^) within a column indicate significant differences at *p* < 0.05. GAE, gallic acid equivalent.

**Table 2:** Maximum wavelength of the UV spectrum (λ_max_), molecular ion ([M − H]^−^) and fragment ions of phenolic compounds identified in purified hawthorn bark extract and its fractions separated on a Toyopearl HW-40S column.

Compound No.	t_R_ (min)	λ_max_ (nm)	[M − H]^−^ (*m*/*z*)	Fragment Ions (*m*/*z*)	Compound
**1**	2.88	278	577	425, 289	B-type procyanidin dimer(1)
**2**	2.99	280	289	–	(+)-Catechin
**3**	3.75	278	577	289	B-type procyanidin dimer(2)
**4**	4.01	278	577	289	Procyanidin B2
**5**	4.10	278	865	577, 289	B-type procyanidin trimer(1)
**6**	4.19	280	289	–	(−)-Epicatechin
**7**	4.22	279	865	577, 289	B-type procyanidin trimer(2)
**8**	4.57	278	577	425, 289	B-type procyanidin dimer(3)
**9**	4.62	280	865	577, 575, 289	B-type procyanidin trimer(3)
**10**	4.95	280	865	577, 289	B-type procyanidin trimer(4)
**11**	5.27	280	865	577, 575, 289	B-type procyanidin trimer(5)
**12**	5.71	268, 348	447	285	Isoorientin
**13**	5.77	280	1153	865, 577, 289	B-type procyanidin tetramer(1)
**14**	5.81	268, 348	447	285	Orientin
**15**	5.87	280	577	289	B-type procyanidin trimer(6)
**16**	6.31	279	577	289	B-type procyanidin dimer(4)
**17**	6.48	268, 336	431	311, 283, 269	Vitexin
**18**	6.59	256, 350	–	301	Quercetin derivative
**19**	6.76	270, 338	431	311, 283, 269	Isovitexin
**20**	7.03	256, 355	463	301	Quercetin 3-*O*-galactoside
**21**	7.12	280	577	289	B-type procyanidin dimer(5)
**22**	7.21	255, 350	463	301	Quercetin 3-*O*-glucoside
**23**	7.37	280	865	577, 575, 289	B-type procyanidin trimer(7)
**24**	7.52	279	863	575, 289	A-type procyanidin trimer
**25**	7.57	278	575	289	A-type procyanidin dimer
**26**	7.75	280	1153	865, 577, 289	B-type procyanidin tetramer(2)
**27**	8.05	280	865	577, 289	B-type procyanidin trimer(8)
**28**	8.50	280	1153	865, 577, 289	B-type procyanidin tetramer(3)

Compound numbers correspond to the peak numbers shown in the chromatogram in Figure 2.

**Table 3:** Content of individual phenolics in purified hawthorn bark extract and its fractions separated on a Toyopearl HW-40S column (mg/g extract or fraction).

Phenolic Class	Compound	Extract	Fraction			
			I	II	III	IV
Flavan-3-ol	(+)-Catechin	2.7 ± 0.3	10.4 ± 0.3	nd	nd	nd
	(−)-Epicatechin	211.9 ± 4.3	741.3 ± 13.7	2.5 ± 0.2	nd	nd
	**Σ Flavan-3-ols**	**214.6 ± 4.1**	**751.7 ± 13.6**	**2.5 ± 0.2**	**–**	**–**
Procyanidin dimer	Procyanidin B2	187.5 ± 4.4	14.3 ± 1.1	770.6 ± 49.7	47.3 ± 2.4	nd
	B-type procyanidin dimer(1)	6.2 ± 0.5	5.8 ± 0.3	53.8 ± 2. 9	nd	nd
	B-type procyanidin dimer(2)	63.7 ± 1.4	nd	17.0 ± 1.1	278.6 ± 12.2	nd
	B-type procyanidin dimer(3)	19.8 ± 1.2	4.7 ± 1.5	7.4 ± 0.4	14.1 ± 1.6	nd
	B-type procyanidin dimer(4)	tr	nd	3.1 ± 0.3	8.2 ± 0.5	nd
	B-type procyanidin dimer(5)	24.4 ± 1.7	nd	4.0 ± 0.2	86.7 ± 3.8	nd
	A-type procyanidin dimer	1.1 ± 0.1	nd	nd	4.9 ± 0.4	nd
	**Σ Procyanidin dimers**	**302.7 ± 6.9**	**24.8 ± 2.7**	**855.9 ± 53.7**	**439.8 ± 20.1**	**–**
Procyanidin trimer	B-type procyanidin trimer(1)	tr	nd	nd	nd	121.0 ± 16.5
	B-type procyanidin trimer(2)	tr	nd	nd	nd	33.5 ± 8.3
	B-type procyanidin trimer(3)	24.1 ± 1.7	nd	nd	30.4 ± 1.9	12.8 ± 2.2
	B-type procyanidin trimer(4)	3.2 ± 0.5	nd	nd	9.9 ± 1.3	11.9 ± 1.1
	B-type procyanidin trimer(5)	100.1 ± 2.4	nd	3.5 ± 0.3	332.0 ± 8.9	185.4 ± 13.8
	B-type procyanidin trimer(6)	15.3 ± 1.1	nd	nd	5.2 ± 0.2	94.7 ± 3.6
	B-type procyanidin trimer(7)	7.3 ± 0.4	nd	nd	nd	20.5 ± 3.6
	B-type procyanidin trimer(8)	tr	nd	nd	nd	14.0 ± 0.0
	A-type procyanidin trimer	tr	nd	nd	15.8 ± 1.0	8.1 ± 0.1
	**Σ Procyanidin trimers**	**150.0 ± 3.2**	**–**	**3.5 ± 0.3**	**393.3 ± 12.7**	**501.9 ± 20.7**
Procyanidin tetramer	B-type procyanidin tetramer(1)	45.9 ± 1.3	nd	nd	nd	314.4 ± 11.4
	B-type procyanidin tetramer(2)	tr	nd	nd	nd	8.3 ± 0.1
	B-type procyanidin tetramer(3)	2.6 ± 0.1	nd	nd	nd	22.8 ± 0.9
	**Σ Procyanidin tetramers**	**48.5 ± 1.2**	**–**	**–**	**–**	**345.5 ± 17.2**
Other flavonoids	Isoorientin	0.7 ± 0.0	2.4 ± 0.2	nd	nd	nd
	Isovitexin	3.9 ± 0.1	14.7 ± 0.5	nd	nd	nd
	Orientin	6.5 ± 0.2	25.6 ± 1.2	nd	nd	nd
	Vitexin	3.1 ± 0.0	12.1 ± 0.3	0.8 ± 0.0	nd	nd
	Quercetin derivative	0.6 ± 0.0	1.9 ± 0.3	nd	nd	nd
	Quercetin 3-*O*-galactoside	10.3 ± 0.1	40.9 ± 2.9	nd	nd	nd
	Quercetin 3-*O*-glucoside	1.1 ± 0.1	4.4 ± 0.5	4.7 ± 0.4	nd	nd
	**Σ Other flavonoids**	**26.2 ± 0.2**	**102.0 ± 5.8**	**5.5 ± 0.4**	**–**	**–**
	**Σ Phenolics**	**742.0 ± 10.7 ^b^**	**878.5 ± 21.6 ^a^**	**867.4 ± 54.5 ^a^**	**833.1 ± 33.1 ^a^**	**847.4 ± 29.4 ^a^**

Results are presented as mean ± standard deviation (*n* = 3). Different superscript letters (^a^ and ^b^) within a row with the sum of phenolics indicate significant differences at *p* < 0.05.

**Table 4:** Antiradical activity against ABTS^•+^ and DPPH^•^, and ferric-reducing antioxidant power (FRAP) of purified hawthorn bark extract and its fractions separated on a Toyopearl HW-40S column.

Extract/Fraction	ABTS^•+^ Scavenging Activity (mmol TE/g)	DPPH^•^ Scavenging Activity (mmol TE/g)	FRAP (mmol Fe^2+^/g)
Extract	8.48 ± 0.28 ^bc^	6.10 ± 0.12 ^b^	17.72 ± 0.81 ^b^
Fraction I	7.86 ± 0.11 ^c^	4.95 ± 0.10 ^c^	13.91 ± 0.73 ^c^
Fraction II	9.28 ± 0.35 ^a^	6.71 ± 0.11 ^a^	21.06 ± 1.15 ^a^
Fraction III	8.97 ± 0.29 ^ab^	6.45 ± 0.08 ^a^	18.27 ± 0.08 ^b^
Fraction IV	8.95 ± 0.12 ^ab^	6.52 ± 0.10 ^a^	17.67 ± 0.44 ^b^

Results are presented as mean ± standard deviation (*n* = 3). Different superscript letters (^a–c^) within a column indicate significant differences at *p* < 0.05. TE, Trolox equivalent; ABTS^•+^, 2,2′-azino-bis(3-ethylbenzothiazoline-6-sulfonic acid) radical cation; DPPH^•^, 2,2-diphenyl-1-picrylhydrazyl radical.

## Data Availability

Data is contained within the article.
